# Bicarbonate Increases Ischemia-Reperfusion Damage by Inhibiting Mitophagy

**DOI:** 10.1371/journal.pone.0167678

**Published:** 2016-12-14

**Authors:** Bruno B. Queliconi, Alicia J. Kowaltowski, Roberta A. Gottlieb

**Affiliations:** 1 Departamento de Bioquímica, Instituto de Química, Universidade de São Paulo, São Paulo, São Paulo, Brazil; 2 Cedars-Sinai Heart Institute, Los Angeles, California, United States of America; Thomas Jefferson University, UNITED STATES

## Abstract

During an ischemic event, bicarbonate and CO_2_ concentration increase as a consequence of O_2_ consumption and lack of blood flow. This event is important as bicarbonate/CO_2_ is determinant for several redox and enzymatic reactions, in addition to pH regulation. Until now, most work done on the role of bicarbonate in ischemia-reperfusion injury focused on pH changes; although reperfusion solutions have a fixed pH, cardiac resuscitation protocols commonly employ bicarbonate to correct the profound acidosis associated with respiratory arrest. However, we previously showed that bicarbonate can increase tissue damage and protein oxidative damage independent of pH. Here we show the molecular basis of bicarbonate-induced reperfusion damage: the presence of bicarbonate selectively impairs mitophagy, with no detectable effect on autophagy, proteasome activity, reactive oxygen species production or protein oxidation. We also show that inhibition of autophagy reproduces the effects of bicarbonate in reperfusion injury, providing additional evidence in support of this mechanism. This phenomenon is especially important because bicarbonate is widely used in resuscitation protocols after cardiac arrest, and while effective as a buffer, may also contribute to myocardial injury.

## Introduction

One of the most fundamental aspects of ischemia-reperfusion (IR) injury is the disruption of blood flow in the tissue. Without circulation, oxygen content rapidly decreases while CO_2_/bicarbonate accumulates. When the tissue is reperfused there is a burst in reactive oxygen species (ROS) production which is responsible for most of the damage [1]. While there are studies that have analyzed the effects of bicarbonate on IR injury [2–6], they do not differentiate between acidosis and bicarbonate concentrations. This can be specially misleading as acidosis can protect the heart against IR independently of bicarbonate [6]. We have shown that increased bicarbonate concentrations can increase IR injury in a pH-independent environment [7], an effect that is important to consider when designing reperfusion strategies.

The role of the CO_2_/bicarbonate pair is not limited to pH regulation. Bicarbonate is necessary for several redox reactions [8–10], for mitochondrial metabolism [11] and acts as a co-transporter in membrane channels [11,12]. While CO_2_/bicarbonate is known to participate in redox metabolism [8,13], we were the first to show that the presence of bicarbonate increases protein oxidation under pathophysiological conditions [7]. The accumulation of the oxidized proteins could arise from increased oxidant production associated with mitochondrial dysfunction, or could be the result of impaired clearance via the proteasome [14], or a failure to clear oxidant-generating mitochondria and oxidized proteins by autophagy, mitophagy or mitochondria-derived vesicles [15,16].

Here we unveil the mechanism that promotes increased toxicity caused by the presence of bicarbonate in ischemia-reperfusion. We show that mitophagy impairment is responsible for the increase in oxidized proteins, while there are no changes in mitochondrial function, oxidant production, proteasome activity or nonspecific autophagy.

## Materials and Methods

Detailed information on methods used on this paper is contained on S1 File on the online support information.

### Materials

All chemicals were of the highest purity available from Sigma (St. Louis, MO, USA), unless otherwise specified. Bafilomycin A was purchased from EMD Millipore (Billerica, MA, USA) and Amplex Red from Molecular Probes (Eugene, OR, USA).

### Mitochondria isolation

Heart mitochondria were rapidly isolated as previously described [17,18]. After the heart was removed it was minced and subsarcolemmal mitochondria were isolated by differential centrifugation.

### Mitochondrial hydrogen peroxide production

Isolated mitochondria were incubated in mitochondrial experimental buffer (in mM: 125 sucrose, 65 KCl, 10 HEPES, 2 inorganic phosphate, 2 MgCl_2_, and 0.01% bovine serum albumin, adjusted to pH 7.2) in the presence of 25 μM Amplex Red and 0.5 U/mL horseradish peroxidase. Hydrogen peroxide production was measured under previously described conditions [18,19].

### Mitochondrial respiration

Respiration of isolated mitochondria was measured in the mitochondrial experimental buffer using a high resolution oxygen electrode (Oroboros), as described before [18].

### Immunostaining and imaging

Immunostaining was done as previously described [20]. Cells were PFA fixed and stained for CoxIV and DAPI. Imaging was done in a Keyence fluorescence microscope using a 100x oil lens.

### Mitochondrial network quantification

Keyence software was used to produce a macro to detect and quantify the mitochondrial particle sizes in an unbiased way. The aspect ratio (AR) and format factor (FF) were quantified using the described formulas, AR = (major axis)/(minor axis) FF = Perimeter/4*Pi*Area^2^.

### Proteasome activity

Proteasome activity was measured by quantifying increasing fluorescence of Suc-Leu-Leu-Val-Tyr-AMC, as described previously [21].

### Isolated heart perfusion

Heart perfusion was conducted as described previously [7,22]. Briefly, hearts were rapidly removed from male anesthetized (pentobarbital sodium 60 mg/kg i.p. and heparin 100 U i.p.) and heparinized Sprague-Dawley rats (~300 g, 2–3 months), and Langendorff-perfused using oxygenated Krebs-Henseleit buffer (described below). Hearts were eliminated from the study if the time between rat death and the beginning of perfusion was longer than 2 min. All studies were conducted in accordance with guidelines for animal care and use established by the *Sociedade Brasileira de Ciência em Animais de Laboratório* and approved by the Animal Care and Use Committee at Cedars-Sinai Medical Center in conformance to the Guide for the Care and Use of Laboratory Animals (National Institutes of Health publication no. 85–23, revised 1996).

After isolation, the hearts were stabilized for 30 min and then subjected to 30 min ischemia followed by 15 min reperfusion. The perfusion used was a modified Krebs buffer containing (in mmol/L) 118 NaCl, 1.2 KH_2_PO_4_, 4.7 KCl, 1.2 MgSO_4_, 1.25 CaCl_2_, 10 glucose, and 20 Na^+^-Hepes, pH 7.4 which was then gassed with pure O_2_, for 0% CO_2_ or gassed with 90% O_2_ + 10% CO_2_ for 10% CO_2_ at 37°C. We confirmed that the pH remained at 7.4 after gassing.

### Creatine kinase quantification

Creatine kinase was quantified in heart perfusate, cell supernatant and total cellular protein using CREATINE KINASE (CK)-SL kit by Sekisui.

### Cardiac HL-1 cell cultures and simulated ischemia/reperfusion (sI/R)

Cardiac HL-1 cells were kindly donated by Professor William C. Claycomb. For routine growth, HL-1 cells were maintained in T-75 flasks at 37°C in an atmosphere of 5% CO_2_ in Claycomb medium (Sigma) supplemented with 0.1 mM norepinephrine, 100 U/mL and 100 U/mL penicillin/streptomycin, 2 mM glutamine, and 10% fetal bovine serum. Experiments were done in 100 mm plates with cells grown to 100% confluence.

sI/R was conducted as previously described [22]. Cells were subjected to 150 min of simulated ischemia in Krebs buffer lacking glucose and supplemented with 5 mM sodium lactate and 20 mM 2-deoxyglucose in a GasPak^™^ EZ Anaerobe Pouch System (Franklin Lakes, NJ, USA) gassed with 100% N_2_ (0% CO_2_) or 90% O_2_ + 10% CO_2_ (10% CO_2_). Simulated ischemia was followed by 5 min of reperfusion with the modified Krebs buffer described above and gassed with pure O_2_ for 0% CO_2_ or gassed with 90% O_2_ + 10% CO_2_ for 10% CO_2_. All cell treatments were performed at 37°C.

### Western blots

Western blots (WB) were done as previously described [7,20]. The samples from hearts and cells were prepared by homogenizing the tissue or the cells in the presence of an extraction buffer (mmol/L, 250 sucrose, 20 Tris-HCl, 2 EDTA, 10 EGTA, pH = 7,5 freshly added protease and phosphatase cocktail), aliquoted in sample buffer and frozen at -80°C until use.

The blots were analyzed using Image J or Image Lab (BioRad) software. Blots were compared to the 0% CO_2_ control or to the 0% CO_2_ ischemic group and normalized by Ponceau staining.

### Statistics

All experiments presented were replicated at least three times, and statistical analysis was conducted using GraphPad Prism 5. For studies with more than 2 groups, 2-way ANOVA was used; when just 2 conditions were compared, Student’s t-test was used. Differences were considered significant if p < 0.05.

## Results

### Bicarbonate increases I/R injury

**Fig 1:**
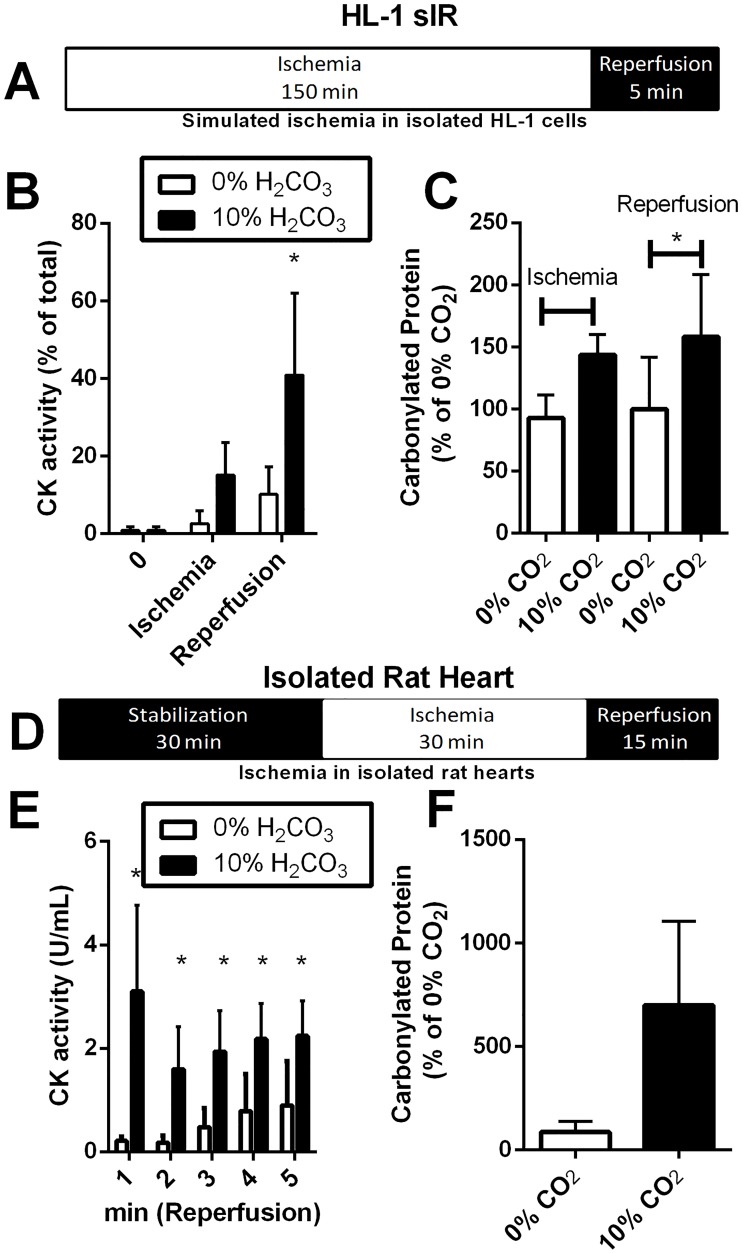
Bicarbonate exacerbates ischemia/reperfusion injury. HL-1 cells were subjected to 150 min ischemia followed by 5 min of reperfusion (A); CK release to the supernatant was measured at the indicated times (B); protein carbonylation content was measured by western blot in the cell lysate before and after the 5 min reperfusion (C). Isolated rat hearts were subjected to ischemia for 30 min and 15 min reperfusion (D); CK release into the perfusate was measured during reperfusion (E); protein carbonylation content was measured by western blot at the end of reperfusion (15 min) (F).

We previously reported that bicarbonate increases ischemia-reperfusion (I/R) injury [7]. To understand the molecular events that lead to the damage increase we used two models of I/R injury: isolated rat hearts subjected to global no-flow ischemia followed by reperfusion, and HL-1 cells subjected to glucose deprivation and hypoxia. In both settings pH was held constant at 7.4. In the cardiac-derived cell line (HL-1 cells), cells were exposed to simulated ischemia-reperfusion injury (sI/R), with an ischemia of 150 min and a reperfusion of 5 min (Fig 1A) to promote damage similar to that found in the isolated heart. Cell death was measured by following creatine kinase (CK) release at the end of the ischemia and after the reperfusion (Fig 1B). The presence of bicarbonate promoted a significant increase in cell death after reperfusion when compared to control conditions. Additionally, the presence of bicarbonate promoted a significant increase in protein carbonylation after 5 min reperfusion when compared to no bicarbonate sI/R (Fig 1C).

We confirmed these results in isolated rat hearts subjected to I/R in the presence (10% of CO_2_) or absence (0% CO_2_) of bicarbonate (Fig 1D). After 30 min stabilization, the hearts were exposed to 30 min of global no-flow ischemia and 15 min of reperfusion, when CK release was measured (Fig 1E). After 15 min reperfusion, the hearts were frozen in liquid nitrogen and protein carbonylation was measured in the protein extract (Fig 1F). There was a significant increase in CK release in the group exposed to bicarbonate, indicating greater injury (Fig 1E). This was paralleled by a trend for increased carbonylation damage (p = 0.0525) in the heart protein extract (Fig 1F). We then proceeded to investigate the molecular mechanism(s) responsible for increased ischemia/reperfusion injury in the presence of bicarbonate.

### Effect of bicarbonate on autophagy

**Fig 2:**
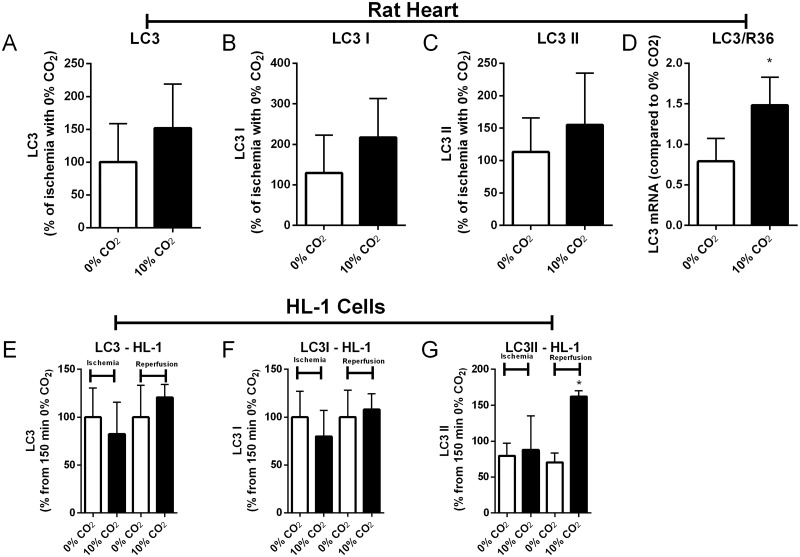
Bicarbonate does not change LC3 expression, but changes LC3 transcription. LC3 was measured by western blot or RT-PCR as described in the Material and Methods. Hearts were subjected to 30 min ischemia followed by 15 min reperfusion, after which protein was extracted to quantify LC3 expression (A-C). In the same treatment, mRNA was isolated and LC3 mRNA was quantified by RT-PCR (D). HL-1 cells were subjected to 150 min ischemia followed by 5 min reperfusion. Cell extracts prepared at the indicated times were probed for LC3 expression (E-G).

Autophagy is one of the main systems responsible for cellular homeostasis and organelle quality control. Therefore, autophagy is in the center of the possible causes for the accumulation of oxidized proteins. We started by measuring LC3 content, an autophagy marker, in rat hearts and HL-1 cells under IR injury in the presence or absence of bicarbonate (Fig 2A–2C and 2E–2G). In the heart, we saw a trend toward an increase in LC3-I and -II, but this did not reach statistical significance; however, there was a significant increase in the concentration of LC3 mRNA (Fig 2D), which could signify a transcriptional response to an unmet need for autophagy [23]. In the HL-1 model, the presence of bicarbonate promoted an increase in the amount of LC3-II (the activated form of LC3) during reperfusion (Fig 2G), which could reflect increased autophagy initiation or impaired flux.

**Fig 3:**
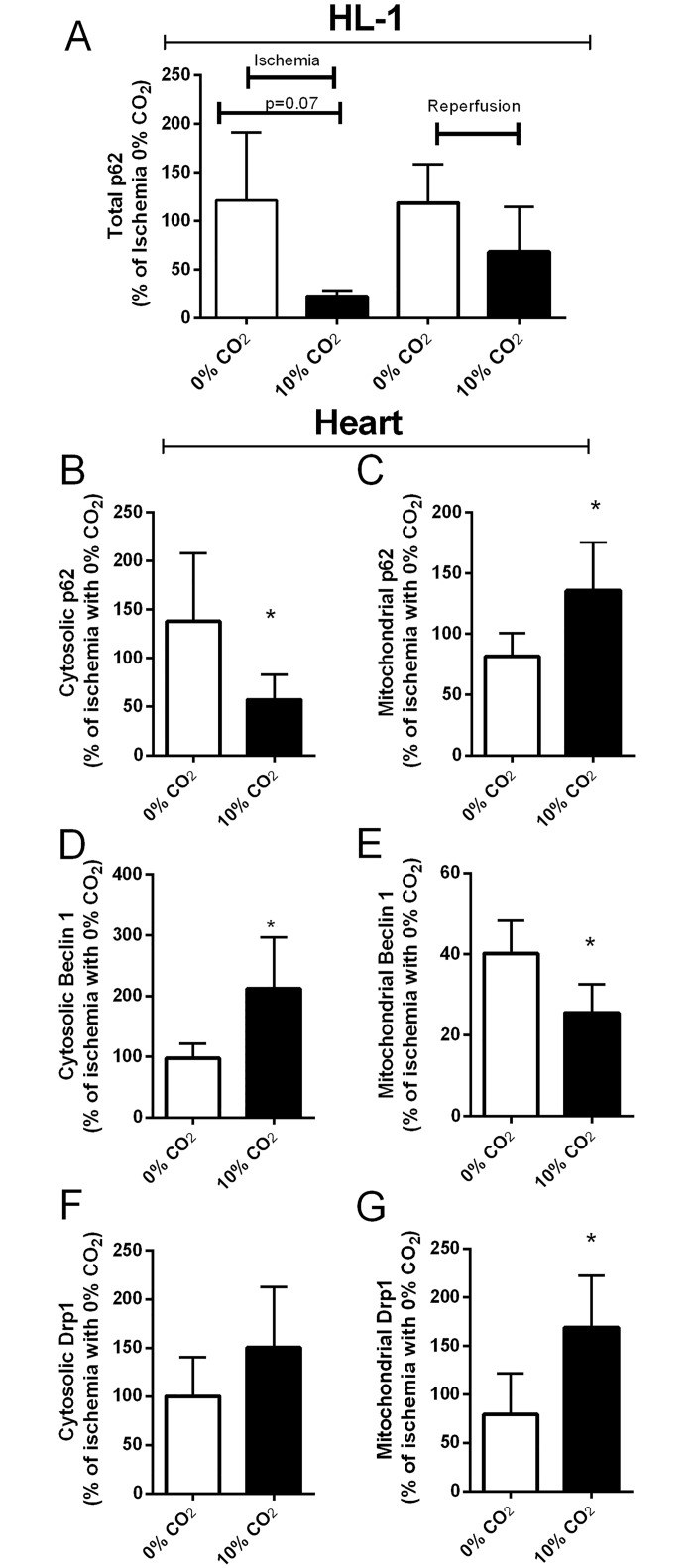
Autophagy markers accumulate in the mitochondrial fraction. HL-1 cells were subjected to 150 min ischemia followed by 5 min reperfusion, and cell extracts were obtained at the indicated times and probed for p62 by western blot (A). Isolated rat hearts were subjected to 30 min ischemia followed by 15 min reperfusion, and the heart homogenates were fractionated by differential centrifugation to yield cytosol (B, D, F) and mitochondria (C, E, G). The resulting fractions were probed for p62 (B and C), Beclin 1 (D and E) and Drp1 (F and G) by western blot.

We looked further into the autophagy machinery by analyzing p62, Beclin 1 and Drp1, three proteins that are part of the autophagy and mitophagy pathway (Fig 3). We initially probed for the amount of p62; the presence of bicarbonate decreased the total and cytosolic (Fig 3A and 3B) concentration of p62 indicating either increased autophagy, which would help to clear oxidized proteins and would therefore be expected to be protective [24,25], or autophagy inhibition, that would lead to increased death [26,27].

### Effect of bicarbonate on mitochondrial autophagy

As mitochondria are an important source of oxidants, their proteins are necessarily a major target of oxidative damage. For that reason, we followed by investigating mitochondrial autophagy, which revealed a different picture from our findings in the cytosolic compartment. In the rat hearts challenged with I/R, we found that the presence of bicarbonate resulted in a greater accumulation of p62 in the mitochondrial fraction (Fig 3C), suggesting that impaired mitophagy might explain the increased damage [28,29]. Bicarbonate also affected the distribution of Beclin 1 and Drp1 (Fig 3D–3G). Whereas Beclin 1 was significantly increased in the cytosol in the presence of bicarbonate, it was decreased in the heavy membrane/mitochondrial fraction. In contrast, Drp1 showed increased accumulation in mitochondria in the presence of bicarbonate. These findings are consistent with intact autophagy induction, but impaired autophagic clearance of mitochondria.

### Proteasome activity

**Fig 4:**
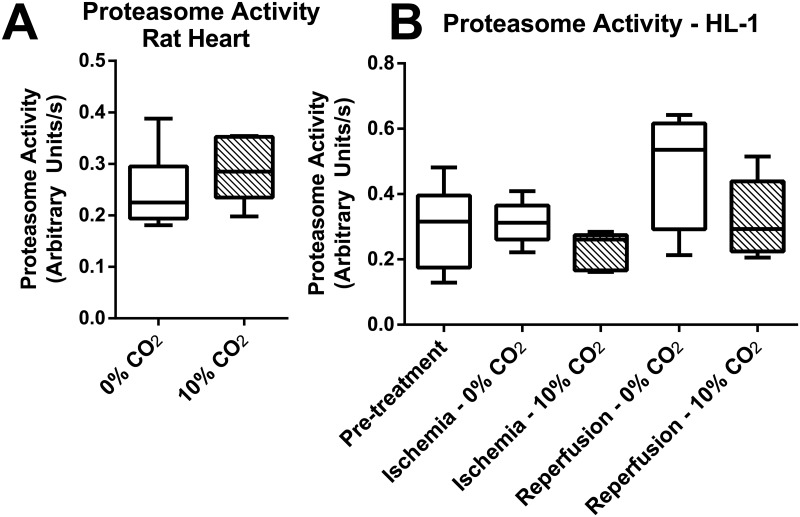
Proteaso me activity is not affected by bicarbonate. Proteasome activity was measured as described in Materials and Methods. Rat hearts were subjected to 30 min ischemia followed by 15 min reperfusion and the proteins were collected and proteasome activity was measured (A). HL-1 cells were subjected to 150 min ischemia followed by 5 min reperfusion; cellular extracts were obtained at the indicated times and proteasome activity was measured (B).

As the proteasome can also degrade oxidized and damaged proteins [14,30,31] we checked proteasomal activity. We measured the activity of the 20S subunit using a fluorescence substrate. Proteasome activity was measured in rat heart lysates (Fig 4A) and in HL-1 lysates (Fig 4B) in the presence or absence or bicarbonate. We did not detect any significant difference in proteasome activity. Nonspecific protease activity detected by MG-123 also showed no difference (data not shown).

### Effect of bicarbonate on mitochondrial phenotype

**Fig 5:**
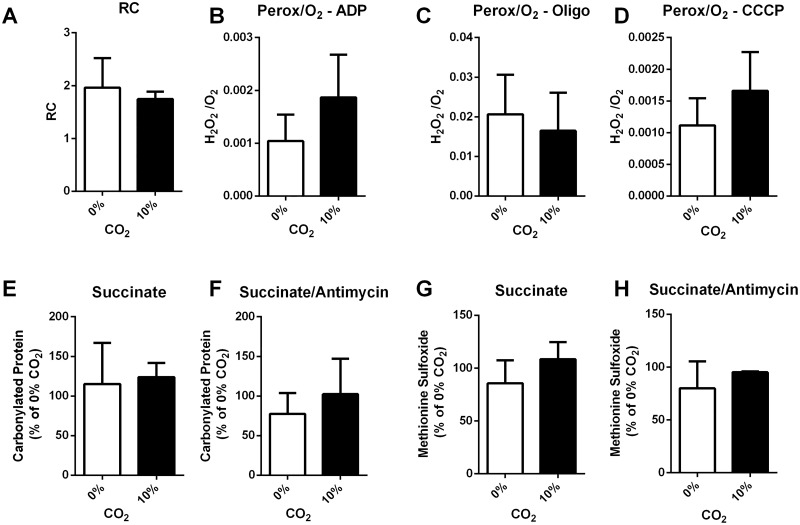
H_2_O_2_ production from isolated mitochondria is not increased by bicarbonate. Mitochondria isolated from rat hearts were subjected to 0 or 10% bicarbonate; hydrogen peroxide production as a function of oxygen consumption was measured in the presence of ADP (A), ADP + Oligomycin (B) or ADP + Oligomycin + CCCP (C). Respiratory control (RC) (D) was measured as described in Materials and Methods. To examine protein oxidative modifications, mitochondria were incubated in media with Succinate (E and G), or Succinate + Antimycin (F and H) for 15 min at 37°C; the mitochondria were then pelleted and protein carbonyl (E and F) and methionine sulfoxide (G and H) content was measured by western blot.

The inhibition of mitophagy observed could be secondary to modifications of the mitochondrial population. To test this possibility, we measured several mitochondrial parameters in the presence or absence of bicarbonate. We started by measuring respiration, membrane potentials, coupling and H_2_O_2_ production from isolated mitochondria from rat hearts incubated with different concentrations of bicarbonate. The presence of bicarbonate had no effect on mitochondrial respiration (S1 Fig) or mitochondrial coupling (Respiratory control—Fig 5A). We next measured H_2_O_2_ production and compared absolute levels (S2 Fig) or levels relative to oxygen consumption under the same conditions (Fig 5B–5D). One important point to be raised is that the method we used is selective for H_2_O_2_; however, the presence of bicarbonate might lead to formation of other oxidants. To verify if other types of oxidants were being generated, we analyzed oxidant-related protein modifications. To create several different oxidant production situations, we incubated isolated mitochondria under different conditions: low (in the presence of succinate) (Fig 5E and 5G) or high (succinate + antimycin) (Fig 5F and 5H). After 15 min incubation, we measured the amount of protein carbonylation (Fig 5E and 5F) and methionine sulfoxide (Fig 5G and 5H). The presence of bicarbonate didn’t affect the amount of protein oxidation, indicating that oxidant levels and modifications caused by them are indeed unaltered.

### Effect of bicarbonate on mitochondrial morphology

**Fig 6:**
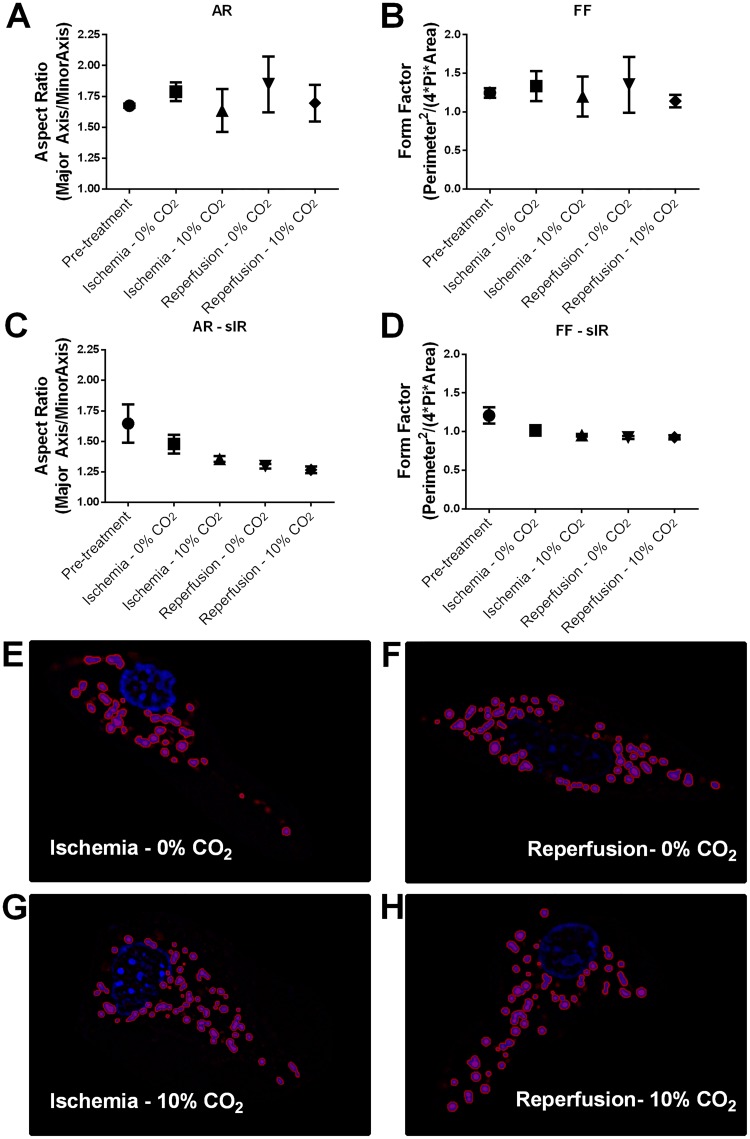
Mitochondrial morphology is not affected by bicarbonate. Mitochondrial morphology under control (A and B) or sI/R conditions (C and D) was measured by calculating their aspect ratio (A and C) (Major axis/Minor Axis) and format factor (B and D) (Perimeter^2^/(Pi*4*Area)). Representative images of mitochondrial morphology analyses using Keyence software; CoxIV is red, DAPI is blue and selected mitochondria are purple (E-H).

Alterations in mitochondrial morphology can also contribute to impaired mitophagy and might contribute to altered oxidant production in intact cells [32–35]. To address this, we performed immunofluorescence imaging of HL-1 cells incubated in the presence or absence of bicarbonate under control (Fig 6A and 6B and S3 Fig) or ischemic conditions (Fig 6C–6H). Ischemia promoted fragmentation of mitochondria, as described before [36], but the presence or absence of bicarbonate did not affect mitochondrial morphology.

### Autophagy inhibition mimics the increase in damage caused by bicarbonate

**Fig 7:**
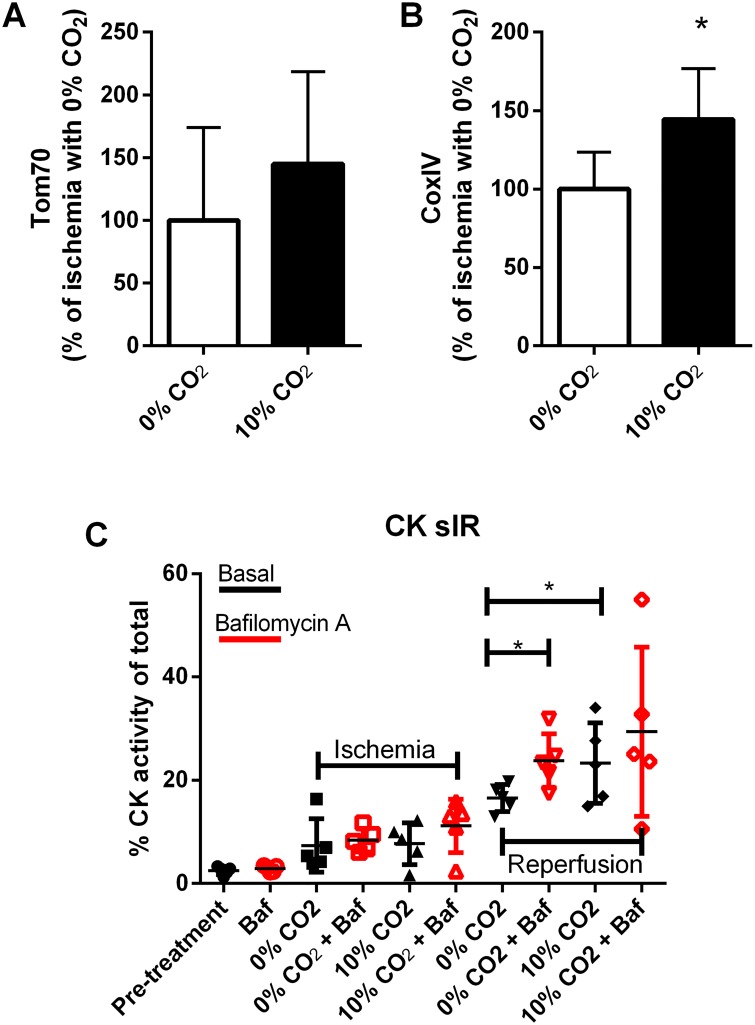
Bicarbonate promotes accumulation of mitophagy cargo and exacerbation of sI/R injury comparable to lysosomal blockade. TOM70 (A) (proteasome degraded) and COXIV (B) (autophagy degraded) were probed by western blot in total extracts from hearts subjected to 30 min ischemia followed by 5 min of reperfusion. HL-1 cells were subjected to 150 min ischemia followed by 5 min reperfusion in the presence or absence of bicarbonate and/or Bafilomycin A (autophagy inhibitor, Baf, red symbols); creatine kinase release was measured at the indicated times (C).

To obtain more solid proof that bicarbonate-mediated inhibition of mitophagy was responsible for the damage observed, we examined mitochondrial proteins that have been described to be degraded by the proteasome (Tom70) or by autophagy (CoxIV) [37,38] (Fig 7A and 7B). Bicarbonate presence resulted in higher levels of CoxIV, whereas changes in Tom70 did not reach statistical significance. These findings are consistent with impaired mitophagy and intact proteasomal function.

Given the evidence for mitophagy inhibition, we next investigated whether global inhibition of autophagic flux could mimic the increase in damage seen by the presence of bicarbonate. We incubated HL-1 cells with or without bafilomycin A (BAF: an autophagic flux inhibitor), while subjecting them to sI/R in the presence or absence of bicarbonate (Fig 7C). The presence of BAF increased I/R injury in the no-bicarbonate group to the same level as the presence of bicarbonate, but BAF did not cause additional injury in the bicarbonate-exposed group. Taken together, these results suggest that in the setting of ischemia/reperfusion, bicarbonate exacerbates injury by interfering with mitophagy, resulting in impaired clearance of oxidatively damaged proteins and mitochondria.

## Discussion

Bicarbonate is the main intracellular buffer, and as such is often viewed as being innocuous, being added to experimental buffers without any consideration regarding possible biological effects [9,11,39]. Indeed, the use of bicarbonate to adjust the pH and its presence in crystalloids used in reperfusion treatment is common norm in clinical settings and accepted as inoffensive. There are several types of reperfusion solutions, with specific pH, varying salts, bicarbonate and other additions, but only a few studies comparing them [40,41]. Particularly in ischemia/reperfusion injury, where bicarbonate content is known to vary considerably [42] and the main component of the damage occurs during reperfusion time [1,43], the solution chosen for reperfusion may greatly affect treatment and results.

We focused here on understanding what molecular events were linked to damage promoted by bicarbonate (Fig 1). Our data describing an increase in damage show the snapshot of the time point most sensitive to damage, the initial reperfusion phase. In this phase, autophagy is necessary to degrade mitochondria so there is no release of cell death-inducing cytochrome c and mitochondrial DNA, that can trigger inflammation and apoptosis [44]. We show that the accumulation of oxidized proteins in the presence of bicarbonate is due the inhibition of mitochondrial autophagy, and not related to increased oxidant production (Fig 5) nor changes in proteasome activity (Fig 4). In fact, mitochondrial autophagy is highly important in cardiac metabolism, helping shape the adult cardiomyocyte [45,46], acting as an essential step in protective ischemic pre-conditioning [47] and heart remodeling [35,48].

**Fig 8:**
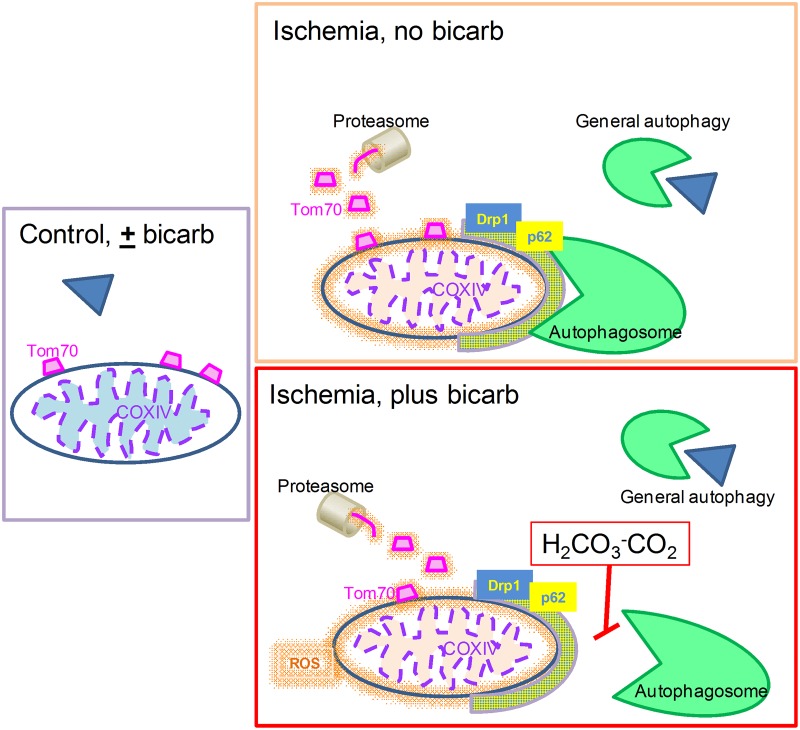
Scheme representing the events that lead to increased damage in I/R and sI/R in the presence of bicarbonate. I/R causes mitochondrial oxidant production with resulting oxidative damage to macromolecules (orange glow). Outer mitochondrial membrane proteins such as TOM70 are degraded by the proteasome, and the damaged mitochondrion is separated from the network by Drp1 and marked for autophagic removal by p62. Bicarbonate interferes with mitophagy, resulting in the accumulation of oxidized proteins, functional impairment, and cell death. Bicarbonate does not affect mitochondrial oxidant production, morphology, proteasome activity, or general autophagy.

Our findings give support to the idea that, despite intact autophagic flux, mitophagy is impaired by the presence of pathologically-relevant levels of bicarbonate. Indeed, when we used Bafilomycin A to block all lysosome-dependent protein degradation, the damage in the absence of bicarbonate was raised to the same level as the one with bicarbonate, while damage with bicarbonate remained unchanged. This suggests that lysosomal inhibition was the cause of the damage promoted by bicarbonate, and clearly indicates that it interferes with mitophagy flux, promoting tissue damage (Fig 8). Overall, our data add to prior findings demonstrating the importance of mitophagy in cardiac protection [28,29,47] by demonstrating that the presence of bicarbonate inhibits mitophagy specifically, causing an increase in the damage triggered by ischemia/reperfusion. Our results uncover a need to reevaluate the use of bicarbonate-containing buffers in clinical resuscitation and myocardial reperfusion protocols.

## Supporting Information

S1 File More detailed Material and methods description is contained on Autophagy-Bicarbonate—Online MM.doc. (DOC)

S1 Fig Mitochondrial respiration is not affected by bicarbonate. Mitochondria isolated from rat hearts were incubated in the presence of the indicated concentration of bicarbonate, and oxygen consumption was measured in the presence of ADP (200 mM).
(TIF)

S2 Fig Absolute mitochondrial ROS production is not affected by bicarbonate. Mitochondria isolated from rat hearts were incubated in different concentration of bicarbonate while hydrogen peroxide production was measured in the presence of Rotenone (A), Antimycin (B), Succinate (C), Succinate+ADP (D), Succinate+ADP+Oligomycin (E) or Succinate+ADP+Oligomycin+CCCP (F).
(TIF)

S3 Fig Mitochondrial morphology is not affected by bicarbonate. **A-D**—Representative images of the cells with mitochondrial and nuclear staining under control conditions. Cells were fixed and stained with CoxIV antibody (red) and DAPI (Blue). Contours show automatic selection by Keyence software and analyzed mitochondrial area.
(TIF)

## Abbreviations

AR: Aspect Ratio
BAF: Bafilomycin A
CK: Creatine Kinase
FF: Format Factor
I/R: Ischemia/reperfusion
ROS: Reactive Oxygen Species
sI/R: Simulated Ischemia-reperfusion
WB: Western blot
