# Supplementary material for: Bicarbonate Increases Ischemia-Reperfusion Damage by Inhibiting Mitophagy
Source: PLoS One. 2016 Dec 14;11(12):e0167678. doi: 10.1371/journal.pone.0167678 (PMC5156406; doi:10.1371/journal.pone.0167678)
Supplement: S1 File — (DOC) [file pone.0167678.s001.doc]

**Online Material**

Detailed Methods

**Mitochondrial isolation**

Heart mitochondria were rapidly isolated as previously described^1,2^. The heart was removed and placed in isolation buffer (in mM; 300 Sucrose, 10 Hepes, 2 EGTA, pH 7.2), at 4°C. The tissue was minced in the presence of 0.5 mg of type I protease (bovine pancreas) to release mitochondria from within muscle fibers and later washed in the same buffer in the presence of 1 mg/mL BSA. The suspension was homogenized in a 40-mL tissue grinder and centrifuged at 800 g for 5 min. The resulting supernatant was centrifuged at 9500 g for 10 min. The mitochondrial pellet was washed and the final pellet was resuspended in a minimal volume of isolation buffer.

**Mitochondrial hydrogen peroxide production**

Mitochondrial H_2_O_2_ release was measured in 0.125 protein/mL suspensions in experimental buffer (in mM; 125 sucrose, 65 KCl, 10 Hepes, 2 inorganic phosphate, 2 MgCl_2_, and 0.01% bovine serum albumin, adjusted to pH 7.2), at 37°C, with continuous stirring. Amplex Red (25 μM) oxidation was followed in the presence of 0.5 U/mL horseradish peroxidase and succinate (1 mM) as substrate. Amplex Red is oxidized in the presence of exogenous horseradish peroxidase bound to H_2_O_2_, generating resorufin, which can be detected fluorimetrically using a fluorescence spectrophotometer operating at 563 nm of excitation and 587 nm of emission^2,3^. Calibration was conducted by adding H_2_O_2_ at known concentrations (A_240_ = 43.6 M^–1^**^.^**cm^–1^). The following drugs were added sequentially: ADP (200 µM), Oligomycin (1 µM), FCCP (1 µM).

**Mitochondrial respiration**

Mitochondrial O_2_ consumption^2^ was monitored in 0.125 mg mitochondrial protein/mL suspensions under the same conditions as H_2_O_2_ release measurements using a computer-interfaced Clark-type electrode (Oroboros) operating with continuous stirring at 37°C.

**Immunostaining and imaging**

Immunostaining was conducted as previously described^4^. Cells were fixed using 4% PFA in PBS for 15 min followed by 3 x 5 min PBS washes. Permeabilization was promoted for 30 min in PBS + 0.25% Triton X-100, followed by blocking in PBS + 0.1% Tween 20 + 1%BSA for 30 min. CoxIV antibody (1:100 in blocking buffer, Cell Signaling - 4844) was incubated overnight at 4°C, followed by 3 x 5 min PBS washes. The secondary antibody (Alexa Fluor® 594 Goat Anti-Rabbit IgG - Invitrogen - A11012 - 1:250 in blocking buffer) was incubated for 1 hour and washed 3 x 5 min PBS washes. 4',6-Diamidino-2-phenylindole (DAPI) (0.1 µg/mL in PBS) was added next for 10 min followed by a PBS wash and imaging using a Keyence fluorescence microscope. The plates were kept in the dark until use and images were obtained using a 100x oil lens.

**Mitochondrial network quantification**

Keyence software was used to detect and quantify the mitochondrial particle sizes. The cells were selected individually and haze and noise reduction were applied using the same standard for all images. The quantification was then carried out using 20 cells per condition; the average of each measurement was considered the experiment measurement. The aspect ratio (AR) and format factor (FF) were quantified by previously described formulas, AR = (major axis)/(minor axis), FF = Perimeter/4*Pi*Area^2^.

**Proteasome activity**

Proteasome activity was measured as described previously^5^. We used a SpectraMax M3 (Molecular Devices) and measured fluorescence (Ex 380 nm/Em 460 nm) every 30 s during 30 min at 37°C. Samples (10 µg) were added to a 100 µl Tris-Mg buffer (50 mM Tris, 5 mM MgCl_2_, pH = 7.5) in the presence of 125 nM Suc-Leu-Leu-Val-Tyr-AMC. Controls using MG123 (125 nM) were run and no nonspecific activity was detected.

**Isolated heart perfusion**

Heart perfusion was conducted as described previously^6,7^. Briefly, after anesthesia and heparinization (pentobarbital sodium 60 mg/kg i.p. and heparin 100 U i.p.) hearts were rapidly removed from male Sprague-Dawley rats (~300 g, 2-3 months), and Langendorff-perfused with oxygenated Krebs-Henseleit buffer (described below). Hearts were eliminated from the study if the time between rat death and the beginning of perfusion was longer than 2 min. All studies were conducted in accordance with guidelines for animal care and use established by the *Sociedade Brasileira de Ciência em Animais de Laboratório (SBCAL)* and approved by the Animal Care and Use Committee at Cedars-Sinai Medical Center in conformance to the Guide for the Care and Use of Laboratory Animals (National Institutes of Health publication no. 85- 23, revised 1996).

After isolation, the hearts were stabilized for 30 min then subjected to 30 min ischemia and 15 min reperfusion. The reperfusion was conducted with buffers containing 0 and 10% CO_2_. The buffer for 0% CO_2_ contained (in mmol/L) 118 NaCl, 1.2 KH_2_PO_4_, 4.7 KCl, 1.2 MgSO_4_, 1.25 CaCl_2_, 10 glucose, and 20 Na^+^-Hepes, pH 7.4 gassed with pure O_2_, at 37°C and for 10% (in mmol/L) 118 NaCl, 25 NaHCO_3_, 1.2 KH_2_PO_4_, 4.7 KCl, 1.2 MgSO_4_, 1.25 CaCl_2_, 10 glucose, and 20 Na^+^-Hepes, pH 7.4, at 37°C gassed with 90% O_2_ + 10% CO_2_.

**Creatine kinase quantification**

Creatine Kinase (CK) quantification was achieved by using a CREATINE KINASE (CK)-SL kit by Sekisui (cat. 326-10). Briefly, aliquots from perfusate, cell supernatants and total cellular protein were stored at 4°C until measurements were made (maximum 3 days without detectable loss of activity), a 5 μl sample was added to 100 µl of reagent and changes in absorbance at 340 nm were followed in 30 s intervals during 30 min. A linear regression was used to quantify CK activity by using the following formula: ΔA = εlΔC, where ΔA = |linear regression slope|, ε = NADH extinction coefficient (6,220 M^−1^cm^−1^), I = optical path and ΔC = Δ [NADH^+^]. The result is expressed considering that 1 unit of enzyme oxidizes 1 µM of NADH per minute.

**Cardiac HL-1 cell cultures and simulated cellular IR**

Cardiac HL-1 cells were kindly donated by Professor William C. Claycomb. These cells maintain their cardiac phenotype during extended passages and present ordered myofibrils, cardiac-specific junctions and voltage-dependent currents that are characteristic of a cardiac myocyte phenotype^8^. For routine growth, HL-1 cells were maintained in T-75 flasks at 37°C in an atmosphere of 5% CO_2_ in Claycomb medium (Sigma) supplemented with 0.1 mM norepinephrine, 100 U/mL and 100 U/mL penicillin/streptomycin, 2 mM glutamine, and 10% fetal bovine serum. Experiments were done in 100 mm plates and with 100% confluence.

Cell simulated IR (sIR) was done as previously described^7^. Cells were submitted to 150 min of ischemia in a GasPak™ EZ Anaerobe Pouch System (Franklin Lakes, NJ, USA) and 5 min of reperfusion. During the whole experiment, the cells were kept at 37°C and in Krebs buffer. To initiate ischemia, cells were placed inside the GasPak bag and the desired gas was pumped inside (filling the bag followed by air removal 10 times; the last time the bag was left with positive pressure to ensure no leakage was present), 100% N_2_ (0% CO_2_) or 10% CO_2_ + 90% N_2_ (10% CO_2_).

Ischemic Krebs buffer had no glucose, was supplemented with 5 mM sodium lactate and 20 mM 2-deoxyglucose and gassed with 100% N_2_ (0% CO_2_) or 90% O_2_ + 10% CO_2_ (10% CO_2_). Reperfusion was promoted in the modified Krebs buffer described above.

**Western blots**

Western blots were conducted as previously described^4^. The samples from hearts and cells were immediately frozen at -80°C after the experimental procedure. Homogenization was performed in extraction buffer (0.2 M Tris-HCl, 2 mM EGTA, 20 mM EDTA, phosphatase inhibitors and 1:10 Sigma proteinase inhibitor cocktail, pH 7.5). Heart tissue homogenization was achieved using a Polytron (PowerGen 125 by Fisher Scientific) (3 x 5 s, with 10 s in ice in between); cells were homogenized by passing through a 28 G needle. Samples were maintained on ice throughout all procedures.

Samples were thawed a single time for homogenization, followed by protein quantification using Bradford. WB aliquots were prepared in sample buffer ready to apply to the gel (kept at -80°C until use). For regular WBs, 30 µg of protein were used per lane; for carbonylation and methionine sulfoxide detection, 5 µg of protein were used per lane. Before running, samples were boiled, cooled over ice and resolved on a gradient gel (Bolt 4-12% Bis-Tris Plus Gel - Cat.BG04120)

For the carbonylation WBs, samples were treated as described before^9^ using the Oxyblot kit from Milipore. Briefly, we added SDS to the samples to reach a final concentration of 12% then reacted the proteins with 2,4-dinitrophenylhydrazine (DNPH) for 30 min in the dark followed by the addition of a neutralization buffer.

All membranes were incubated in primary antibody overnight (4°C, rocking), followed by 3 x 20 min wash in TBST (room temperature, rocking). Secondary antibodies were incubated for 1 hour (room temperature, rocking) followed by 3 x 20 min wash in TBST (room temperature, rocking). Clarity Western ECL substrate was used to develop the membrane and the image was captured using Molecular Imager ChemicDoc XRS+ with Image Lab Software from Biorad. The exposure time varied depending on the antibody, and was always less than 5 minutes.

Antibody dilution and catalog numbers were: anti-methionine sulfoxide (1:5000, Oxford Biochemistry - MS01), anti-LC3 (1:2000, Cell Signaling - 4108), anti-p62 (1:2000, Abcam - ab56416), anti-Beclin 1 (1:2000, Cell Signaling - 3738), anti-Drp1(1:2000, Millipore - ABT-155), anti-Tom70 (1:2000, Proteintech - 14528-1-AP) and anti-CoxIV (1:2000, Cell Signaling - 4844).

The blots were analyzed using Image J or Image Lab (BioRad). Blots were compared to the 0% CO_2_ control or to the 0% CO_2_ ischemic group and normalized by Ponceau staining.

**Statistics**

All experiments presented were replicated at least three times, and statistical analysis was conducted using GraphPad Prism 5. Panels A, B, D and E from Figure 2 were analyzed using 2-way ANOVA, while all other data were analyzed using Student t-tests. Correlations were analyzed using linear fits. Differences were considered significant if p < 0.05.

**References**

1. Queliconi BB, Wojtovich AP, Nadtochiy SM, Kowaltowski AJ, Brookes PS. Redox regulation of the mitochondrial K(ATP) channel in cardioprotection. *Biochim. Biophys. Acta* 2011;1813(7):1309-15.

2. Tahara EB, Navarete FDT, Kowaltowski AJ. Tissue-, substrate-, and site-specific characteristics of mitochondrial reactive oxygen species generation. *Free Radic. Biol. Med.* 2009;46(9):1283-97.

3. Zhou M, Diwu Z, Panchuk-Voloshina N, Haugland RP. A stable nonfluorescent derivative of resorufin for the fluorometric determination of trace hydrogen peroxide: applications in detecting the activity of phagocyte NADPH oxidase and other oxidases. *Anal. Biochem.* 1997;253(2):162-8.

4. Andres AM, Hernandez G, Lee P, et al. Mitophagy is required for acute cardioprotection by simvastatin. *Antioxid. Redox Signal.* 2014;21(4):1960-1973.

5. Da Cunha FM, Demasi M, Kowaltowski AJ. Aging and calorie restriction modulate yeast redox state, oxidized protein removal, and the ubiquitin-proteasome system. *Free Radic. Biol. Med.* 2011;51(3):664-70. doi:10.1016/j.freeradbiomed.2011.05.035.

6. Queliconi BB, Marazzi TBM, Vaz SM, et al. Bicarbonate modulates oxidative and functional damage in ischemia-reperfusion. *Free Radic. Biol. Med.* 2013;55:46-53. doi:10.1016/j.freeradbiomed.2012.11.007.

7. Perry CN, Huang C, Liu W, Magee N, Carreira RS, Gottlieb R a. Xenotransplantation of mitochondrial electron transfer enzyme, Ndi1, in myocardial reperfusion injury. *PLoS One* 2011;6(2):e16288.

8. Claycomb WC, Lanson N a, Stallworth BS, et al. HL-1 cells: a cardiac muscle cell line that contracts and retains phenotypic characteristics of the adult cardiomyocyte. *Proc. Natl. Acad. Sci. U. S. A.* 1998;95(6):2979-84.

9. Nakamura A, Goto S. Analysis of protein carbonyls with 2,4-dinitrophenyl hydrazine and its antibodies by immunoblot in two-dimensional gel electrophoresis. *J. Biochem.* 1996;119(4):768-74.
