# Supplementary figures and images for: Bicarbonate Increases Ischemia-Reperfusion Damage by Inhibiting Mitophagy
Source: PLoS One. 2016 Dec 14;11(12):e0167678. doi: 10.1371/journal.pone.0167678 (PMC5156406; doi:10.1371/journal.pone.0167678)

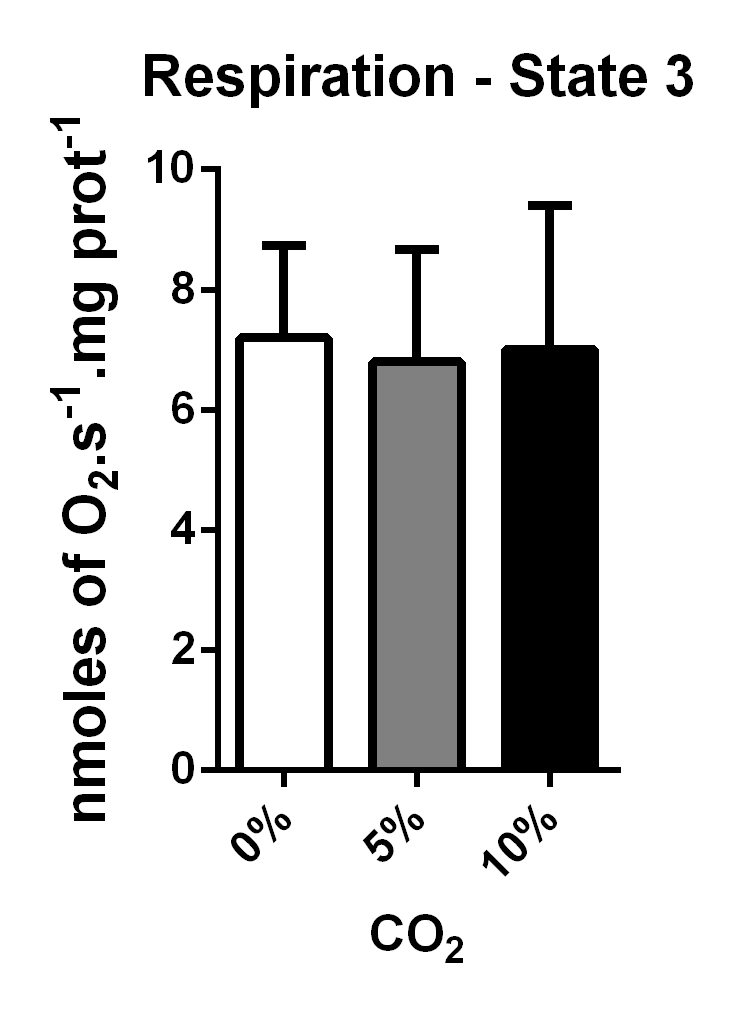

Supplement: S1 Fig — Mitochondria isolated from rat hearts were incubated in the presence of the indicated concentration of bicarbonate, and oxygen consumption was measured in the presence of ADP (200 mM). (TIF) [file pone.0167678.s002.tif]

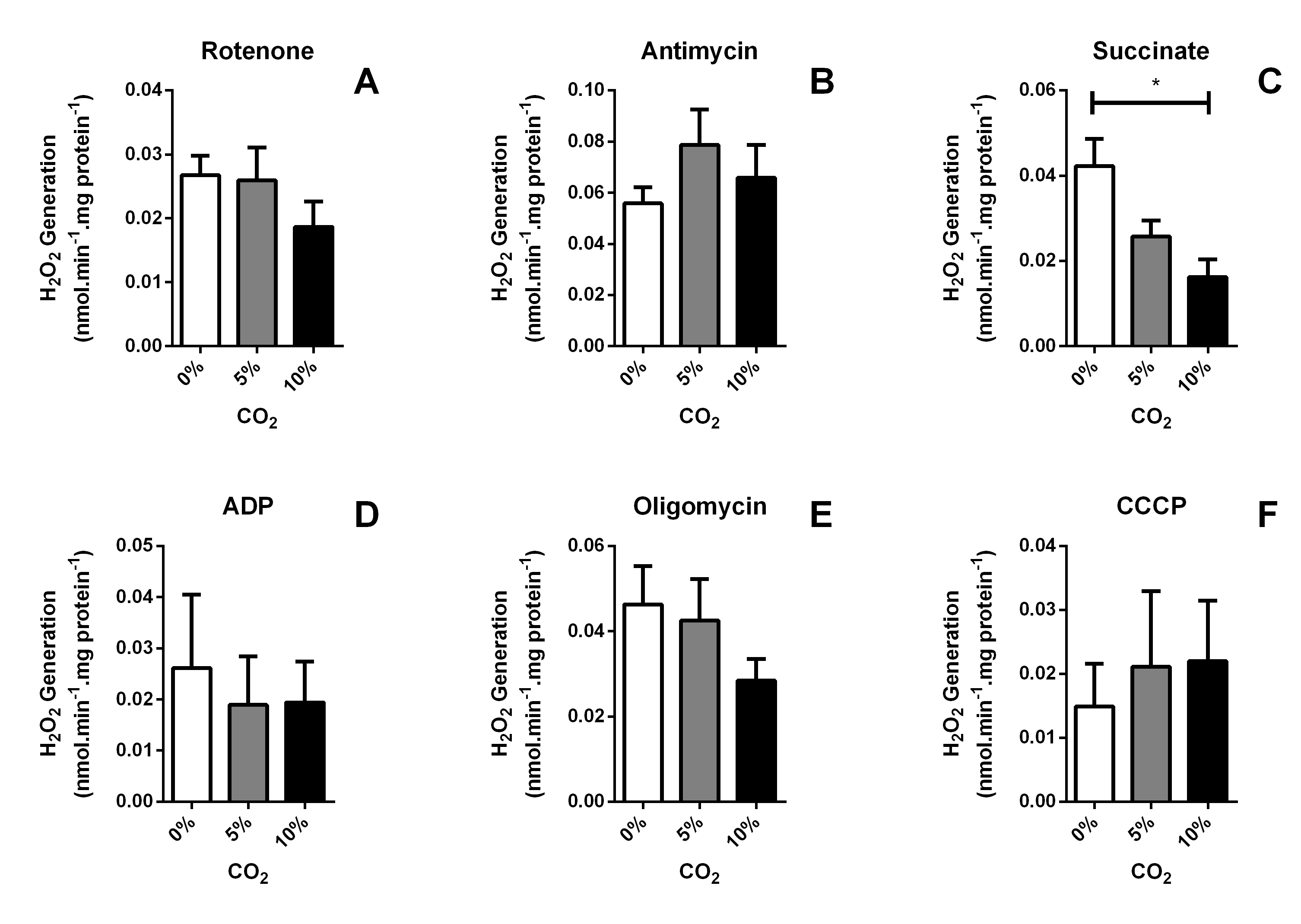

Supplement: S2 Fig — Mitochondria isolated from rat hearts were incubated in different concentration of bicarbonate while hydrogen peroxide production was measured in the presence of Rotenone (A), Antimycin (B), Succinate (C), Succinate+ADP (D), Succinate+ADP+Oligomycin (E) or Succinate+ADP+Oligomycin+CCCP (F). (TIF) [file pone.0167678.s003.tif]

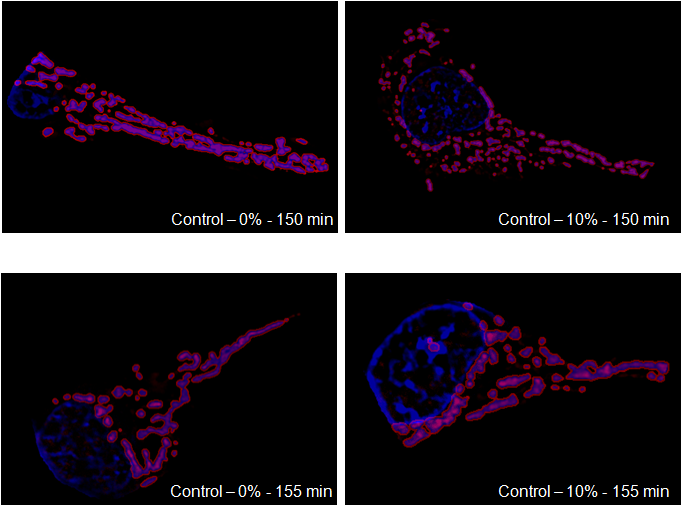

Supplement: S3 Fig — A-D—Representative images of the cells with mitochondrial and nuclear staining under control conditions. Cells were fixed and stained with CoxIV antibody (red) and DAPI (Blue). Contours show automatic selection by Keyence software and analyzed mitochondrial area. (TIF) [file pone.0167678.s004.tif]
